# Oral colonization by *Candida* species in orthodontic patients before, during and after treatment with fixed appliances: A prospective controlled trial

**DOI:** 10.4317/jced.57565

**Published:** 2020-11-01

**Authors:** Icíar Sanz-Orrio-Soler, Santiago Arias de Luxán, Chirag C. Sheth

**Affiliations:** 1 Department of Dentistry, Faculty of Health Sciences, Universidad Cardenal Herrera, CEU Universities, Moncada 46113, Valencia, Spain; 2 Department of Medicine, Faculty of Health Sciences, Universidad Cardenal Herrera, CEU Universities, Moncada 46113, Valencia, Spain

## Abstract

**Background:**

Orthodontic treatment with fixed appliances is associated with changes in oral microbiota, including increased *Candida* colonization. The *Candida* fungus can cause oral lesions and infections such as candidiasis and angular cheilitis, and is harmful to both the patient and the orthodontist. Poor hygiene facilitates the colonization of these microorganisms. The key aim was to quantify the colonization of *C. albicans* in patients prior to beginning orthodontic treatment, and during the treatment process.

**Material and Methods:**

A total of 124 patients (43 males and 80 females) with a mean age of 19.5 years, who required treatment with metal or aesthetic (ceramic) braces, were studied. Microbiological samples were taken from the oral cavity using the swab technique throughout the treatment and cultured on a Sabouraud Dextrose Agar plate and, if positive, cultured on a CHROMagar® Candida plate.

**Results:**

In contrast to other published studies, no statistically significant increase in *C. albicans* colonization was observed during the orthodontic treatment. The fixed appliances had no influence on the presence, absence or level of colonization by *C. albicans* and there were no significant differences between the different appliances studied.

**Conclusions:**

Our study showed that frequency of oral hygiene measures by study participants did not affect the rate of oral carriage of *Candida* in a statistically significant manner. This observation contrasted with published literature, which suggests that thorough brushing is important to prevent the build-up of *Candida* species.

** Key words:**Orthodontics, fixed appliances, oral microflora, Candida albicans.

## Introduction

The goal of all orthodontic treatment is to improve the patient’s occlusion, function, mastication, aesthetics, comfort, self-esteem and overall health (1). However, we must consider that, as with many other interventions, there is potential to cause significant damage to hard and soft tissues, since the type of orthodontic appliances and their components contribute to plaque retention. The plaque build-up is associated with gingival inflammations and periodontal lesions (2,3). Oral pathologies associated with orthodontic treatment arise due to irritating, frictional and traumatic damage, which result in reactive lesions such as frictional hyperkeratosis, traumatic ulcers, traumatic fibroma, fibrous hyperplasia and mucocele. Other oral pathologies associated with orthodontic treatment include herpes and angular cheilitis, which affect the gums and mucous membranes (1).

Angular cheilitis is caused by a variety of microorganisms, amongst which the fungus **Candida* albicans* (*C. albicans*) is a leading cause. It has been observed that orthodontic treatments alter salivary pH, increase the accumulation of dental plaque and make proper oral hygiene difficult, thereby facilitating fungal overgrowth and development of cheilitis (4). Differences between orthodontic appliances contribute to differences in oral colonization and associated pathologies. Aesthetic (ceramic) braces represent a more ecological, microbe-friendly, porous niche than metal braces, and therefore we observe a higher incidence of *Candida* in the former as compared to the latter. (5)

Given the importance of successful orthodontic treatment in the rehabilitation of functional and aesthetic dental and skeletal defects, it is essential to control and reduce the impact of factors that could cause lesions in both the mucosa and the periodontium. Constant observation and comprehensive clinical examination are necessary before, during and after treatment, and it is essential to ensure that the patient is committed to maintaining an extremely high level of oral hygiene in order to achieve a satisfactory final result with no adverse effects and full patient satisfaction.

The main objective of this study is to quantify the colonization of *C. albicans* in patients prior to beginning orthodontic treatment, and during the treatment process. In this study, there are three secondary objectives. These are: analysis of the presence of different strains of *Candida* in the oral cavity, the level of oral colonization by *C. albicans* based on the type of orthodontic appliances worn by the patient, and the volunteers’ level of oral hygiene. The analysis of these factors will allow us to determine the level of risk of *Candida* infection associated with orthodontic appliance use.

## Material and Methods

The project was approved by the Ethics Committee for Biomedical Research of CEU Cardinal Herrera University with file number CEI19/080.

-Volunteer selection

For this study, all patients who started orthodontic treatment with metallic or aesthetic (ceramic) fixed braces between October 2013 and September 2016 in three private orthodontic clinics in Valencia, Spain, were selected for inclusion. The proposed design was a prospective controlled trial.

The inclusion criteria were adult patients who were to undergo treatment with fixed appliances, without previous orthodontic treatment, non-smokers and not suffering from any systemic disease (hematological, nutritional or immunocompromised). In addition, we also excluded patients allergic to nickel (18,19) to eliminate any circumstances that could lead to errors in the results.

Before the fixed appliances were fitted, the patients completed a questionnaire related to their hygiene habits and signed an informed consent form to take part in this study. Subsequently, these documents were stored in accordance with the provisions of the Data Protection Act.

The sample size for this study was calculated using the online sample size calculator, Clincalc (6). The enrolment ratio between the groups was 1 and patients of both sexes were included. The power of the study was 80% and the Alpha error value was 0.05 (6). The study was designed to have two independent working groups with continuous feedback. In order to calculate the sample size, based on previous literature, we estimated the expected mean presence of *C. albicans* in the group with cosmetic fixed appliances as 1.97 ± 0.53 and the comparison group, with metal fixed appliances having 1.36 ± 0.44 (7). The calculations determined that the optimum study size should include two groups of participants, with 54 members per group. One of the major threats to validity of prospective controlled trials is the loss of participants to follow-up. Therefore, to counteract a potential loss of patients, we planned to recruit an additional 15% over the minimum required number. Thus, a total of 62 volunteers were required and recruited for each group.

-Clinical sampling protocol 

An oral microbiological sample was taken from the oral cavity using a sterile swab (Ref. 551291 in Francisco Soria Melguizo S.A.), rubbing it once across the upper vestibule and again over the lower vestibule from right to left, ending at the commissures of the mouth. This process was carried out immediately prior to the fitting of the multibracket fixed appliances, without any type of appliance having been previously worn in the mouth.

The sampling frequency was as described below:

T0: Prior to starting orthodontic treatment.

T1: 1 month after the start of treatment.

T2: 6 months after the start of treatment.

T3: 1 year after the start of treatment 

T4: 6 months after completion of orthodontic treatment.

-Microbiological analysis protocol

Sample handling and enumeration of *Candida*: After taking the sample, the sterile swabs were placed in tubes with 1ml of transport medium. These tubes were stored at 4ºC until processing (never more than 24h). Processing involved streak inoculation of the swab on Sabouraud Dextrose Agar (SDA) plates, followed by incubation at 37ºC for 48 hours, according to manufacturer’s instructions. Grown colonies were tested by germ tube assay to confirm the presence of *Candida* species. Following incubation, the plates were photographed for evidence and the number of colonies counted.

Characterization of *Candida* species: In order to characterize the clinical strains of *Candida*, the swab as streak inoculated on CHROMagar™ *Candida* plates (Becton Dickinson) and cultivated at 37ºC for 48 hours according to manufacturer’s instructions. Following incubation, the plates were photographed for evidence and the species identity of the colonies arising was determined by cross-referencing with the type-strains as published by the manufacturer.

This process was repeated at each sampling timepoint for samples from each of the study volunteers.

-Statistical analysis

The data were analyzed using SPSS v23 software. *Candida* colonization and characterization was carried out and comparisons were made across the 5 sampling time points and between the two orthodontic devices used amongst the study volunteers. Parametric statistics were used except when the number of events analyzed was below 30, when nonparametric statistics were used, specifically the Mann-Whitney test for comparing two independent samples.

We determined 95% CI for proportion estimation, and given the high sample size, we assumed normality (Moivre’s theorem, as a particular case of the Central Limit Theorem). The t-Student test was used to compare means between 2 independent groups, using Levene’s test to assess the equality of variances. For the comparison of *Candida* colonization between orthodontic devices, a repeated ANOVA measurement was used, with the Bonferroni test for multiple comparisons. In order to analyse the dependence between quantitative variables, linear regression with determination of the Pearson R correlation coefficient was used, and for the analysis of the dependence between categorical variables, Pearson’s chi-squared test was used.

For all analyses, a *p*<0.05 was considered significant.

## Results

Table 1 shows the frequency of isolation of *Candida* in the various samples. At timepoint T4, it was not possible to obtain the sample from 13 volunteers.

**Table 1:**
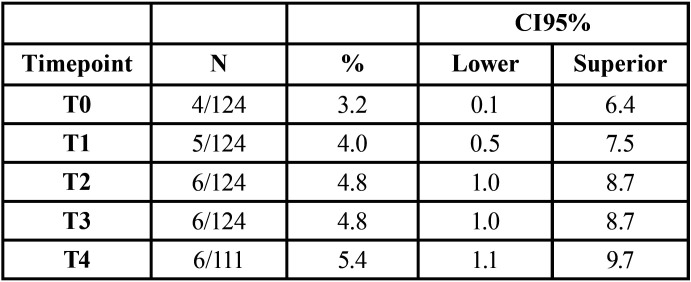
Overall colonization levels by *Candida* at the five sampling time points.

We found no statistically significant difference in the frequency of isolation of *Candida* from the mouths of volunteers across the different time points. Additionally, the 95% confidence intervals overlap completely, so it cannot be said that one or more of the samples are different from the others.

In Table 2, we see the different species at study timepoints 0-4. The most frequently isolated organism was *Candida albicans*. *C. glabrata* and *C. krusei* were each found in 1 volunteer respectively. No *Candida* was isolated from the majority of volunteers at each timepoint. The results showed a slight increase in *Candida* colonization from timepoint 0 (3.2%) to timepoint 4 (4.8%), however this variation was not found to be statistically significant.

**Table 2:**
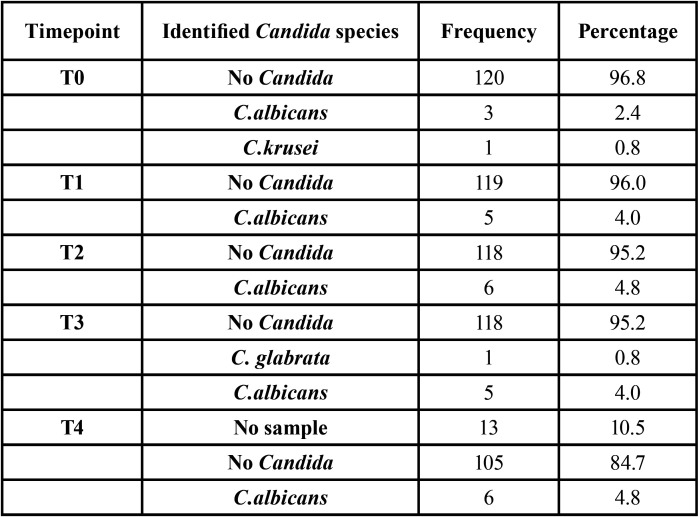
Distribution of *Candida* species at the five sampling time points.

In Table 3, we see the presence of *Candida* in the two types of fixed appliance. There was no statistically significant difference (*p*=0.558) in prevalence between the two appliance types.

**Table 3:**
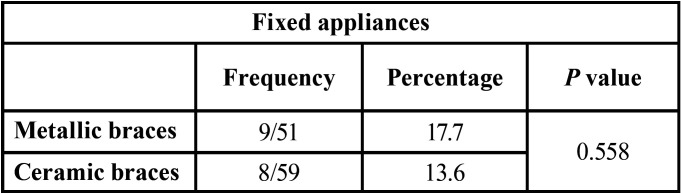
Overall prevalence of *Candida* according to the type of orthodontic appliance.

The results of the initial questionnaire on volunteer’s oral hygiene were cross-referenced with the presence or absence of *Candida* in the oral cavity during the study. A numerical average was calculated for each of the patients (higher values indicate better hygiene), based on their level of hygiene. There were no statistically significant differences associated with the presence of *Candida* (Table 4).

**Table 4:**
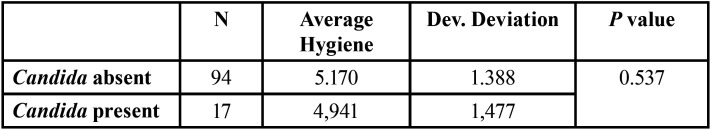
Influence of hygiene on the presence or absence of *Candida* in the oral cavity of study volunteers.

## Discussion

-Patient selection

Previous studies show that removable orthodontic appliances had a lower impact on the oral flora than fixed appliances, with a greater oral carriage and increased prevalence of oral pathologies in patients with the latter type of treatment (4,8-10).

In this study, the exclusion criteria are very similar to other published papers, the most important being smokers (3,11-14). Patients taking prescribed medication such as antibiotics or immunosuppressants (3,4,9-17), as well as those who have previously received orthodontic treatment (9,12) were also excluded as these factors have been shown to cause alterations in oral colonization by *Candida*.

-Sample collection method.

Various techniques have been successfully used for the isolation and identification of *Candida* in oral samples, including saliva culture (3,4,10,11) and swab culture (9,12,14-16,20). We used swab culture given its reliability and simplicity in taking the sample.

As this phase depended on the collection of samples obtained from collaborating clinics, the aim was to facilitate their participation, as it was essential to have as many patients included in the study as possible. Being an easy to use and quick method for both the doctor and the volunteer, it consumed little time and made it easier for both the clinic and the patient to collaborate.

Other authors require volunteers to go 1-2 hours without eating, drinking or brushing their teeth (3,4,11,17) or even a whole day taking no oral hygiene measures, before taking saliva samples (15). These parameters were not taken into account when carrying out this study, but it should be noted that the stated conditions were used in conjunction with the salivary culture technique and not swab culture as in our study.

The duration of follow up and sampling time points found in most studies were: prior to starting treatment, at one month, and at six months (10,12,17).

Previous studies showed that there were changes in oral flora within one month of the braces being fitted (12,21). Lucchese (2018) recorded significant alterations in oral microbiota one month after starting orthodontic treatment (8). It was therefore considered appropriate to take the second sample (T1). None of the studies carried out a sample at the immediately following the orthodontic treatment, so it was decided to carry out a sample six months after completion and thus compare the results.

-Microbiological method of analysis

Many studies limit their analysis of *Candida* colonization to total counts from SDA plate culture, but do not take into account the *Candida* species present (4,10-15,22). Other authors identified the *Candida* species in other ways: culture on corn flour agar and the API 20C method. The first two were for the cultivation of *C. albicans* only and did not identify the different species of *Candida*. For this reason, we decided to rule out these cultivation methods. In contrast, with the API 20 C method, the yeast must first be isolated and then identified using the same method, i.e. a two-step identification process. In addition to being less efficient, the differences with Chromagar *Candida*, where identification is done in one-step, are not statistically significant (4).

-Prevalence of *Candida*

The prevalence of *C. albicans* appears to be very high in the articles reviewed when compared to this study, where it initially appears at only 4.1%. It should be kept in mind that the sample in the current study is a very small proportion of the population and does not reflect the general population, but rather a reduced sample of people seeking orthodontic treatment for functional and aesthetic improvement. In addition, individuals who come to an orthodontic clinic in Spain for treatment often have above-average oral hygiene, which influences their initial oral health.

Arslan *et al.*, in their study on a Turkish population, showed a 58.5% (42/72 patients) prevalence of *Candida* before starting orthodontic treatment. This high percentage may be due to the limited sample size and the health and oral hygiene education conditions in the country concerned. The average age of the volunteer providing the sample in this study, was 19.8 years (12).

Similarly, Dar-Odeh *et al.* in Jordan found a 48% (39/81) prevalence of *Candida* before the start of treatment, of which the majority (17/39) was *C. albicans*. Zheng *et al.* in their study conducted in China, found an incidence of *Candida* in adolescents of 14%, (17) and Hernandez-Solis found it to be 15% (9). This may be due to the fact that patients brushed their teeth before the sample was taken and were not told that they should have spent the previous 2 hours without performing any oral hygiene. Hägg, in Hong Kong, found a prevalence of 30%, 7% and 22% depending on the technique used, with culture by absorption offering a lower percentage (7%) and less consistent with the other techniques used (oral rinse and pooled plaque), which offer more similar results (30% and 22%) (13).

When comparing other studies to the present work, we found a very low presence of *C. albicans* in untreated volunteers at the beginning of treatment and an initial quantitative increase in oral carriage in orthodontic treatment patients (not statistically significant). From this, it can be deduced that the placement of fixed appliances may favor an appearance or increase of *C. albicans*, although more research is required. A study from the University of Bergen (Norway) suggests that excess composite around the braces may cause a build-up of plaque which could also influence the development of *Candida* (23).

The results of the current project are not in line with this theory since no significant increase of *C. albicans* was observed in the treated patients, which may be due to patients’ good awareness of their oral hygiene. It must be recognized that as part of the standard treatment protocol, the collaborating clinics worked hard on conveying on the importance of correct oral hygiene both for health in general and for the proper completion of the treatment. Oral hygiene is one of the most important factors associated with the prevalence of *Candida*, although there are no clinical studies linking it to orthodontic treatment (24). Furthermore, the presence of fixed appliances influence the quality and quantity of oral microbiota, but this is a transitory effect, which depends on oral hygiene control (2). It is highly recommended to establish daily hygiene procedures and individual professional hygiene in all orthodontic patients before starting orthodontic treatment (8).

It is important to remember that increased *Candida* colonization in patients does not mean that they will develop candidiasis, but there will be an increased risk of infection if their immune system has been impaired by other factors such as the use of antibiotics or local trauma due to some kind of device (24).

-Strengths and weaknesses of the study

This is a study with a high sample size, since in each group we obtained samples from more than 50 volunteers and according to the sample size calculation, 28 patients would have been enough. We consider that this gives more weight to the study. Another factor to consider is that all of the cultures were produced by a single researcher (I.S.S.), which leads to fewer errors in the methodology of the study.

Another factor to take into account is that most of the volunteers who began the study continued throughout the research process, with a minimal dropout rate. In addition, in all the studies which were analysed, we verified that the time horizon of the tests performed was limited to a short period, with the latest sample collected in most of the articles reviewed being 6 months after the start. Considering that this study period was excessively short, in the present study, a follow-up of the patient was carried out for a period of up to 6 months after the end of the treatment.

One weakness of this paper was that the patient was asked to answer a questionnaire about their oral hygiene only at the beginning of the treatment, i.e. before the fixed appliance was fitted. This leads to different results from the various other papers. The error occurred because it was a multicentre study. It was estimated that the questionnaire referred to in the various saliva sampling sessions could have been repeated, given that when a patient begins orthodontic treatment, they are strongly urged to follow the hygiene instructions, and even at their monthly check-ups they are given a hygiene check. Taking this into consideration, there could have been a case where a patient’s hygiene habits were regular at the beginning of the treatment, and during the treatment there may have been a considerable improvement and a greater concern for their oral health. In addition, this questionnaire could have been further reinforced or complemented with some objective evidence on the level of hygiene. This could have been a measurement of dental plaque or gingival and periodontal status using quantitative or measurable indices such as Silness and Löe’s plaque or gingival index (20), or Greene and Vermillion’s plaque index (22), since a questionnaire is still a subjective tool, and in this way the response would have been compared objectively. These weaknesses are not considered to give rise to a sensitive method error, and do not invalidate the value of the study, given the guarantees introduced in the selection, size and characteristics of the sample, the duration and in the method of analysis used.

## Conclusions

The presence of *C. albicans* in patients before beginning orthodontic treatment was 3.2%. The fixed appliances had no influence on the occurrence of *C. albicans* and there were no statistically significant differences between the different appliances studied. Neither did the level of hygiene influence its occurrence, but according to the systematic review carried out, it is important not only to brush teeth, but also to brush the tongue, gums and mucous membrane. It is also important to maintain strict hygiene practices in order to avoid the accumulation of *Candida* species.

Proper oral hygiene control is very important for patients, since the appearance of *C. albicans* can cause oral infections that lead to physical discomfort and aesthetic problems. In addition, it can lead to a higher financial cost for medication and treatment of the infection.

Orthodontic instruction must be continuous and consistent. In all visits the patient should be reminded to exercise extreme oral hygiene, even more so in the case of those who are careless or negligent, warning them of the risks they run if they persist with improper oral hygiene. Otherwise, if an infectious disease occurs or continues, the treatment could be delayed, even leading to its interruption and failure.
